# Use of Video Laryngoscope in Sedated Spontaneously Breathing Patients with Predicted Difficult Tracheal Intubation and Impossibility of Using Fibreoptic Bronchoscopy

**DOI:** 10.1155/2021/5524240

**Published:** 2021-04-29

**Authors:** Alba Piroli, Ida Marsili, Franco Marinangeli, Silvia Costanzi, Luca Gentili, Antonella Paladini

**Affiliations:** ^1^Department of Life Health and Environmental Sciences, University of L'Aquila, Piazzale Salvatore Tommasi n. 1, L'Aquila 67100, Italy; ^2^San Salvatore Teaching Hospital of L'Aquila, Via Lorenzo Natali n. 1, L'Aquila 67100, Italy

## Abstract

Intubation with a flexible fibrobronchoscope in an awake patient is frequently considered the technique of choice in patients with predicted difficult intubation. There are, however, situations in which the use of the fibrobronchoscope is not applicable, particularly due to problems attributable to the patient or to limited use of the instrument. In such situations, the video laryngoscope can be a useful alternative, as long as it is associated with adequate sedation of the patient. In fact, it ensures excellent viewing of the glottis, allowing for successful orotracheal intubation to be performed even in case of difficult airways, while keeping the patient spontaneously breathing throughout the procedure. From the data present in the literature, this technique seems to ensure a success rate and a safety profile similar to those obtained with the fibrobronchoscope, moreover, with greater ease of use by the anaesthesiologist. The main purpose of this work is to provide a valid and safe alternative to intubation with a fibrobronchoscope while awake in those patients with anticipated difficult airway management and in whom, for different reasons, fibrobronchoscope cannot be used.

## 1. Introduction

In the case of predicted difficult airway intubation (PDI) in a collaborating patient, the method considering the gold standard is intubation of a sedated patient under spontaneous breathing, using a flexible fibrobronchoscope [1–4]. However, intubation with a fibrobronchoscope can be a difficult technique to learn for anaesthesiologists and requires continuous practice to maintain the ability to use the instrument [5]. In some cases, moreover, the positioning of an endotracheal tube (ETT) through fibrobronchoscopy is difficult and/or impossible, due to problems attributable both to the instrument limitations and to the presence of anatomical alterations of the patient's upper airways. In these cases, it is necessary to resort to an alternative intubation technique, which allows the procedure to be completed successfully while ensuring the safety and comfort of the patient. The video laryngoscope (VLS) can be a useful tool to perform intubation while the patient is awake and when difficulty is predicted and the fibrobronchoscope cannot be used. On the other hand, it is an increasingly widely used tool, also because it is easy for anaesthesiologists to maneuver who thus learns to use it very quickly [6, 7]. Several studies can be found in the literature that compare the use of the fibrobronchoscope vs. the VLS in sedated patients under spontaneous breathing with anticipated PDI, which show, for the most part, a similar performance in the two methods with respect to the percentage of success and safety [5, 8]. The VLS is, therefore, an established alternative to fibrobronchoscope for awake patient intubation in clinical practice.

This current work will proceed to describe three cases of patients with anticipated PDIs, for which the use of a fibrobronchoscope was not possible but was successfully intubated, after sedation, thanks to the use of a video laryngoscope.

### 1.1. Case 1

A 74-year-old male, weight 70 Kg, with an American Society of Anaesthesiology (ASA) physical status II, scheduled for thoracic paravertebral schwannoma exeresis. Preoperative examination revealed ankylosing spondylitis, with fixed neck, complete rigidity of the cervical spine, a Mallampati score of 3, El Ganzouri risk index test of 7, and STOP-Bang score of 3. Patient history described a previous intubation with fibrobronchoscope while awake. Considering the surgical access to the thoracic area, an orotracheal intubation (OTI) with a double lumen tube was necessary, which could not be performed via a fibrobronchoscope. Intubation was, therefore, planned by VLS with the patient awake and sedated.

### 1.2. Case 2

**Figure 1:**
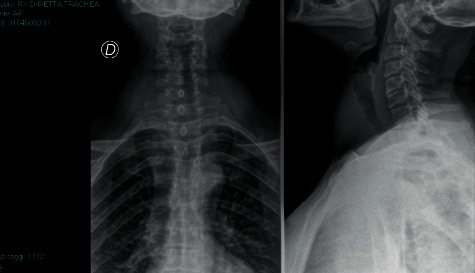
Direct trachea X-ray (Case 2).

A 55-year-old female, weight 65 Kg, with an ASA physical status II, scheduled for total thyroidectomy for multinodular goiter. Airway evaluation revealed a Mallampati score of 2, El Ganzouri risk index test of 2, and STOP-Bang score of 2 with no history of respiratory or swallowing problems. Preoperative cervico-mediastinal radiography showed a marked reduction of the tracheal lumen (Figure 1). Such condition could have made airway management difficult once the patient was asleep. The subsequent CT scan estimated a tracheal diameter of 7 mm in the cervico-thoracic area. A preoperative fibrobronchoscopic examination in the operating room was thus planned to confirm the diagnostic data; this would have required the use of a small-calibre ETT, not compatible with the available fibrobronchoscope.

### 1.3. Case 3

A 64-year-old male, weight 98 kg, with an ASA physical status III, candidate for gastrectomy surgery + distal esophagectomy for adenocarcinoma of the cardia. Preoperative examination revealed a Mallampati score of 3, El Ganzouri risk index test of 4, STOP-Bang score of 6, neck diameter 48 cm, and receding chin, with anticipated difficult ventilation and intubation. In this case as well, the need to use a dual lumen tube to exclude the right lung during surgery made it impossible to use a fibrobronchoscope to perform orotracheal intubation. For this reason, intubation by VLS was scheduled on a sedated patient.

## 2. Methods

All patients, previously informed, provided written informed consent to the proposed procedure. After monitoring routine vital parameters and the level of sedation by Bispectral Index (BIS) and the Ramsey Sedation Scale (RSS), patients were premedicated with atropine 0.5 mg and midazolam 2 mg by IV. An infusion of remifentanil 0.10 mcg/kg/min was started, while keeping the patients under spontaneous breathing with administration of O_2_ 3 Litres/min by nasal cannula; 10 minutes after the start of sedation, local anaesthesia was started, progressively atomizing the oropharynx, the hypopharynx, and the laryngeal aditus with 2% lidocaine (10 ml) using a special cannula (MADgic-Teleflex®); such maneuver needed an additional 10 minutes. When the degree of sedation reached the required levels (BIS 75–80; RSS 3–5) and topical anaesthesia was applied on all the areas involved, after denitrogenation, the glottis was viewed by video laryngoscope (Glidescope®) and subsequently OTI was performed in the first patient by placing a right double lumen tube Ch.41 (Rusch Bronchopart®); in the second patient, a spiral tube (Covidien®) n^o^ 5.5 was placed and, in the third patient, a left double lumen tube Ch.41 (Rusch Bronchopart®) was used. The induction of general anaesthesia was thus carried out.

## 3. Results

**Figure 2:**
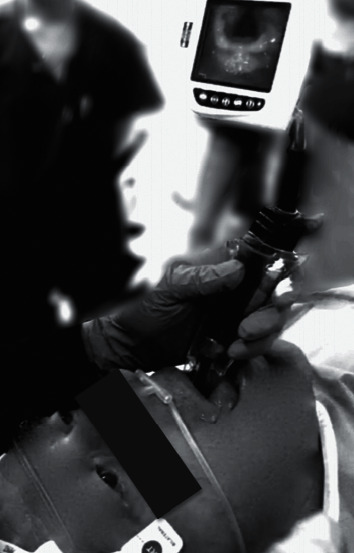
View of glottis with video laryngoscope (Glidescope®). The sedated patient under spontaneous breathing (Case 1).

No complications were reported during the procedures, the haemodynamic parameters remained stable, and there was no reduction in the SaO_2_. In the first and third cases, intubation was successful at the first attempt, with an execution time of 70 sec and 75 sec, respectively; in the second patient, two attempts were necessary, since the initially prepared spiral tube was too large in calibre (n^o^ 6); on the second attempt, a 5.5 calibre tube was successfully positioned in a total time of 84 seconds. Patients tolerated the ETT in an optimal manner, without movements, coughs, or grimaces from pain (Figure 2); when interviewed after 24 hours, none of the three patients remember the procedure and all were extremely satisfied with the method.

## 4. Discussion

Difficulty in ventilation and/or intubation can be a cause of serious harm to the patient, with the possibility of brain damage or death [9–12]. For this reason, a specific and careful preoperative evaluation is necessary of all patients who are candidates for elective tracheal intubation, in order to highlight any difficulties in airways management and to take the necessary precautions [13, 14]. For a long time, intubation with flexible fibrobronchoscope in sedated patients under spontaneous breathing was considered the technique of choice to face predicted difficult intubation situations [1–4, 15]. The use of the fibrobronchoscope, however, poses number of problems for anaesthesiologists, both as regarding learning of the technique used and maintaining the acquired ability over time. In some cases, moreover, the positioning of an endotracheal tube with the aid of the fibrobronchoscope is impossible due to limits of use linked to the instrument or to the particular anatomical condition of the patient. In recent years, use of the VLS has been increasing, and it is progressively becoming the preferred instrument for anaesthesiologists to perform difficult intubations, so as to dramatically modify both the practice of airway management and the very definition of difficult intubation [16]. It offers some undeniable advantages over the fibrobronchoscope, such as a wider view of the airways, which allows better viewing of nearby structures [17–19]; the absence of limitations in the diameter of the tube to be used, with the possibility of also positioning small-calibre tubes; and finally, the greater ease in changing the tracheal tube size, when it becomes necessary, while constantly maintaining a view of the airways [5].

On the other hand, it is quite common to find evidence of failed intubations with the aid of the fibrobronchoscope [20] or of intubations carried out under video laryngoscopic viewing after intubation with a fibrobronchoscope failed [17, 21].

From the data found in the literature, when compared, the two methods are more or less identical as regarding the success rate in OTI execution and safety profile. Moreover, some studies have shown that intubation time would be shorter with the use of a video laryngoscope compared to a fibrobronchoscope [5].

In the cases, we have described, the VLS allowed us to successfully complete the intubation of three patients with anticipated PDI, while maintaining spontaneous breathing throughout the procedure, which could not be intubated with the aid of a fibrobronchoscope: in one patient, due to the narrowing of the tracheal lumen, which required the use of an endotracheal tube of a size too small for the instrument available; in the other two cases, due to the need for positioning of a dual lumen tube, a maneuver that could not be performed with a fibrobronchoscope.

The procedure adopted, ensuring adequate sedation of the patients and effective local anaesthesia, thanks above all to the malleable cannula that allows for a precise and targeted administration of the anaesthetic, has proven, at least in these particular situations, the potential superiority of the VLS compared to the fibrobronchoscope. Moreover, overcoming some of the use limitations of the latter instrument and also showing the total satisfaction of patients in reference to the technique used. Further clinical experience will be needed to confirm such data.

## Additional Points

These cases underwent normal routine surgery. It was therefore not necessary to receive Ethics Committee approval, but all the procedures were in line with the Ethics Indications of the Teaching Hospital ‘‘San Salvatore” for the clinical activities.
